# A Rare Coexistence of Simultaneous Cardio-Cerebral Infarction

**DOI:** 10.1155/2023/9986712

**Published:** 2023-04-13

**Authors:** Vijay Yadav, Ratna Mani Gajurel, Chandra Mani Poudel, Paras Thapa, Manju Sharma, Suraj Shrestha

**Affiliations:** ^1^Department of Cardiology, Manmohan Cardiothoracic Vascular and Transplant Center, Institute of Medicine, Kathmandu, Nepal; ^2^Department of Radiology, Tribhuvan University Teaching Hospital, Kathmandu, Nepal; ^3^Maharajgunj Medical Campus, Institute of Medicine, Kathmandu, Nepal

## Abstract

**Background:**

Contemporaneous acute myocardial infarction (AMI) and acute ischemic stroke (AIS), termed cardio-cerebral infarction (CCI), is a rare medical emergency. The effectual management of this situation is exigent since early management of one condition will inevitably delay the other. *Case Presentation*. A 60-year-old woman presented to our hospital with concurrent AMI of the inferior left ventricular wall, complicated by cardiogenic shock and transient complete heart block, and AIS of more than 4.5 hour duration. The cerebral computerized tomography angiography revealed a right-sided terminal internal carotid artery (ICA) occlusion, and the coronary angiogram depicted double vessel disease with a culprit lesion in the right coronary artery (RCA). The patient underwent mechanical thrombectomy for the ICA occlusion by an interventional neuroradiologist followed by the primary percutaneous coronary intervention of the culprit RCA by the interventional cardiologists in the same setting.

**Conclusion:**

A patient with concurrent AMI and AIS is a challenging situation to treat in the emergency department, and the treatment must be individualized for each patient.

## 1. Introduction

Acute myocardial infarction (AMI) and acute ischemic stroke (AIS) are known to be the leading causes of morbidity and mortality worldwide [1]. Cardio-cerebral infarction (CCI)—a simultaneous occurrence of AMI and AIS within 48 hours—is a rare phenomenon with a reported incidence of 0.009% [2]. Similarly, the in-hospital incidence of AIS after AMI ranges from 0.7% to 2.2% [3].

Owing to a narrow therapeutic time window in both entities (AMI and AIS), CCI requires early diagnosis and intervention and hence, carries high morbidity and mortality [4, 5]. The speedy and efficacious management of CCI is challenging such that the acute management of one at the expense of the other may result in permanent irreversible disability from the infarcted area that receives a delayed intervention. We hereby report a case of 60 years female who presented with AMI first and later developed AIS after 2 hours of the onset of chest pain.

## 2. Case Report

**Figure 1:**
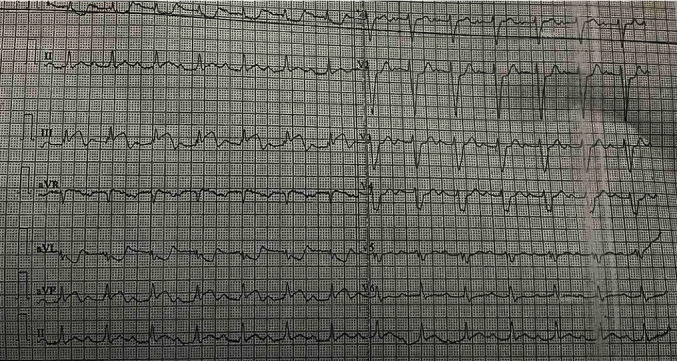
ECG shows ST elevation in inferior leads with first degree heart block.

**Figure 2:**
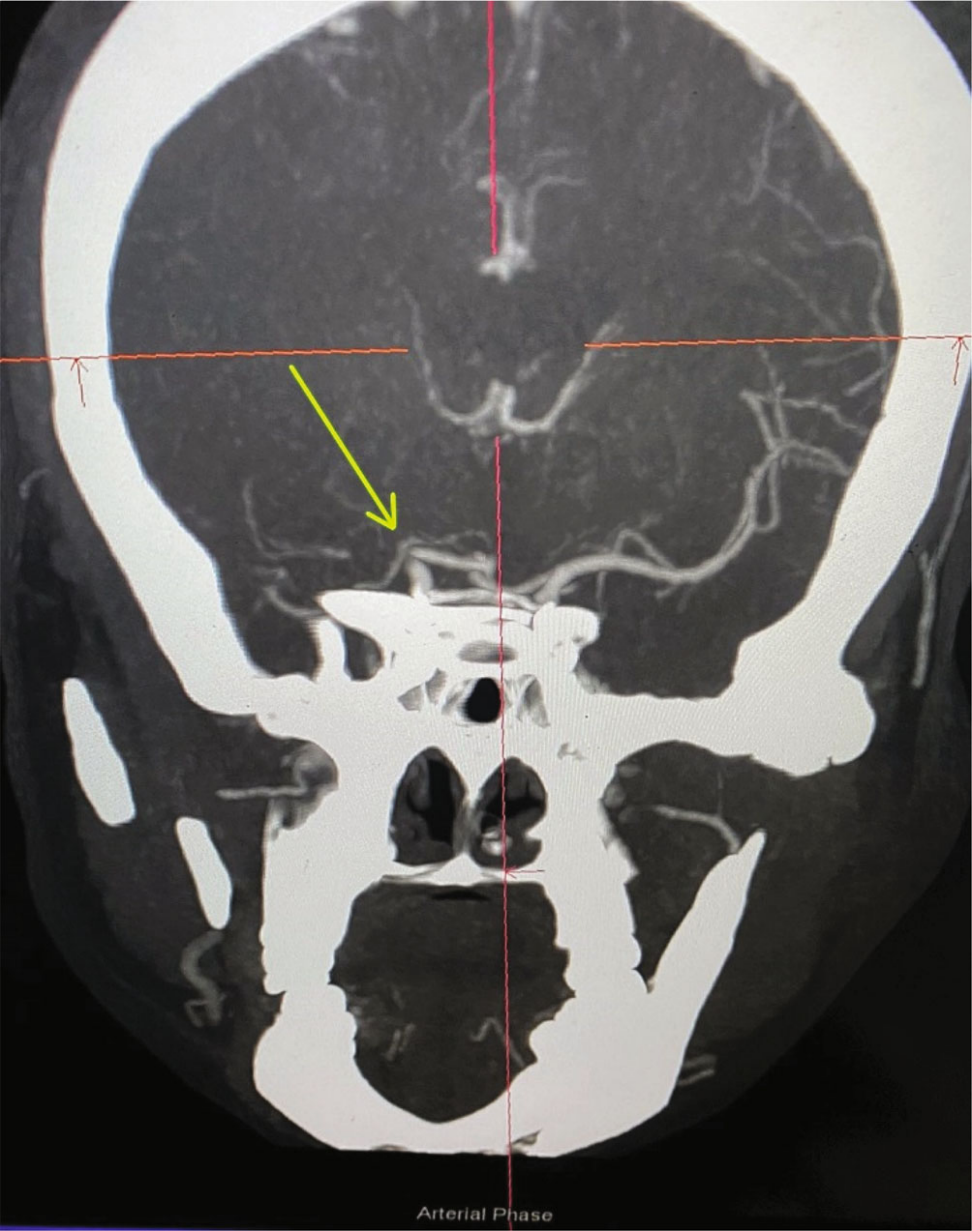
Cerebral CT angiography showing non-opacification of distal ICA and MCA (arrow).

A 60-year-old woman presented to our emergency department with complaints of angina chest pain for the last 8 hours and sudden-onset left-sided hemiparesis with dysarthria, facial deviation, gaze deviation to the right side, and altered mental status for the last 6 hours. She was under medications for systemic hypertension and diabetes mellitus. She had undergone a coronary artery bypass surgery 12 years back with left internal mammary artery to left anterior descending artery (LIMA to LAD) and reverse saphenous vein graft to first diagonal (rSVG to D1). The physical examination revealed a blood pressure of 80/60 mmHg and a regular heart rate of 128 bpm. However, the electrocardiogram (ECG) displayed in the bedside monitor showed transient atrioventricular dissociation with complete heart block. Her Glasgow Coma Scale (GCS) was 13/15 (E3V4M6). The stroke team was consulted, and the National Institutes of Health Stroke Scale score was calculated to be 12. Her baseline ECG in the emergency department depicted ST-segment elevation in the inferior leads and a first-degree heart block (Figure 1). The computerized tomography (CT) scan of the head revealed an ischemic infarct in the right middle cerebral artery (MCA) territory. The cerebral CT angiography showed terminal internal carotid artery (ICA) occlusion on the right side (Figure 2). The serum troponin I level was 11.2 ng/ml (normal value <0.120 ng/ml). The echocardiography showed hypokinesia of basal, mid, and apical inferior and infero-septal walls of the left ventricle with an ejection fraction of 40%. The pharmacological management of AMI started with dual antiplatelets (aspirin 300 mg and clopidogrel 300 mg), rosuvastatin 40 mg, and noradrenaline for cardiogenic shock.

**Figure 3:**
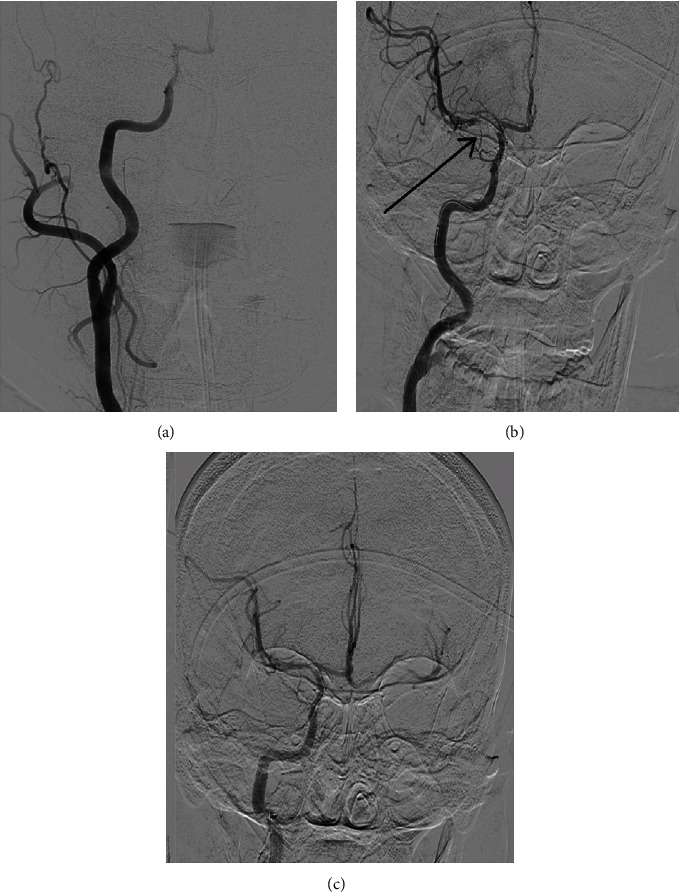
CT cerebral angiogram shows (a) distal ICA occlusion with non-visualization of M1 MCA, (b) stentriever in situ with left ICA run showing a filling defect in distal ICA proximal M1 MCA (arrow), and (c) complete recanalization of ICA MCA.

The mechanical thrombectomy was preferred by the neurologist since the therapeutic window time period for thrombolysis of AIS was over and hence, in accordance with the neurologist's expert advice, an interventional neuroradiologist was immediately consulted, and the patient was shifted to a catheterization laboratory for mechanical thrombectomy. The right femoral approach was preferred, and an 8 French (8F) femoral sheath was inserted over the artery. A bolus of 5000 units of intravenous (IV) unfractionated heparin (UFH) was given immediately after the right femoral puncture. A right common carotid artery angiogram was taken, which confirmed the terminal right-sided ICA occlusion (Figures 3(a) and 3(b)). The guiding catheter-neuron max (0.088″) was parked at the distal cervical ICA. The distal access catheter (DAC) was taken to the ophthalmic segment. A microcatheter (rebar) was used with microwire traxcess (0.014″) and the site of occlusion was crossed. A subsequent angiogram from the microcatheter showed the distal end of the microcatheter in the proximal M2 segment. Eventually, stentriever Solitaire X measuring 4 mm × 40 mm was deployed following which we waited for 5 minutes. It was then slowly taken out while maintaining aspiration on the DAC. A total of two passes were taken and the final angiogram showed complete recanalization of the right ICA, MCA, and its branches [thrombolysis in cerebral infarction (TICI) 3 recanalization; Figure 3(c)]. After waiting for 10 minutes, the final angiogram was taken which confirmed the patency of the right ICA and MCA. Finally, the guide catheter and DAC were taken out. Throughout the procedure, the patient received additional 2000 units of IV UFH infused with 0.9% normal saline.

**Figure 4:**
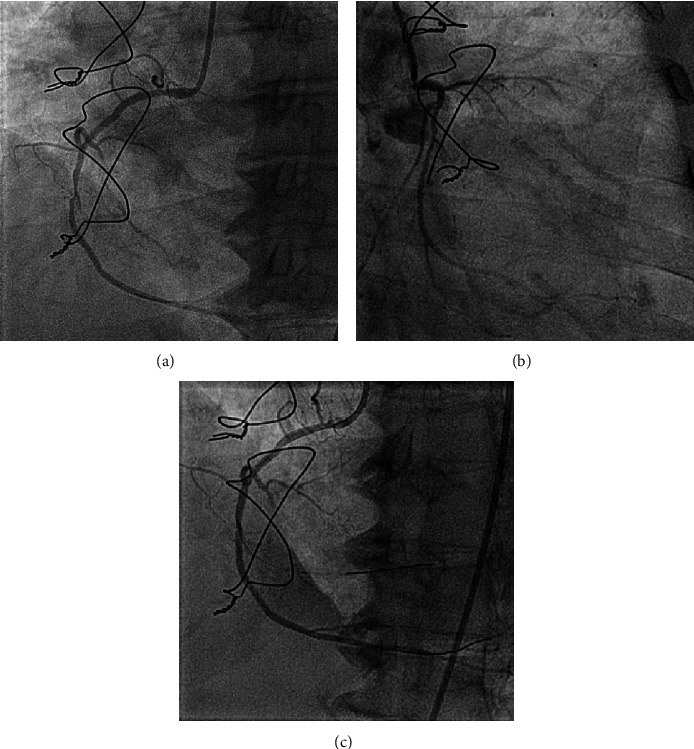
CAG showing (a) diffusely diseased RCA with maximum stenosis of 80–90% at the mid part with 100% occlusion of AM, (b) CTO of LAD from the mid part and small caliber Left circumflex artery, and (c) TIMI 3 flow after RCA revascularization.

In the same setting, a coronary angiogram (CAG) was performed which showed chronic total occlusion of LAD from the mid part and diffusely diseased right coronary artery (RCA) with maximum stenosis of 80–90% at the mid part. It was also noted that she had two large acute marginal (AM) branches—the first had 90–95% focal stenosis, and the second was 100% occluded (Figures 4(a) and 4(b)). CAG of the graft vessels showed patent LIMA to LAD and rSVG to D1 grafts. It was decided to revascularize the culprit right coronary system and eventually, a second intervention was done in the RCA by the interventional cardiologists. A 6F Judkins right was taken for a selective RCA angiogram. Coronary wires, such as ASAHI SION Blue and Balance Middle Weight (BMW) were used to wire RCA and second acute marginal branch (AM-2), respectively. Plain old balloon angioplasty was done in AM-2 with the help of NC QUANTUM PTCA 2.0 mm × 8 mm coronary balloon which was inflated up to a pressure of 14 atmospheres (atm). A sirolimus (ORSIRO) drug-eluting stent (2.75 mm × 35 mm) was deployed at mid-RCA and was additionally post-dilated with an NC QUANTUM PTCA 2.75 mm × 12 mm coronary balloon at 16 atm. A final angiogram confirmed thrombolysis in myocardial infarction (TIMI) flow of 3 in both arteries (Figure 4(c)). Any additional IV UFH was not given during the coronary angioplasty because the activated clotting time was adequate from the previously infused UFH during the mechanical thrombectomy. The CHB resolution was followed by RCA angioplasty, and hence, the pacemaker insertion was not done. Unfortunately, the patient passed away on the following day due to the development of a massive intracranial hemorrhage with a significant midline shift that eventually led to cardio-pulmonary arrest.

## 3. Discussion

We present a rare case report of CCI from Nepal, which was managed simultaneously with percutaneous interventions for the respective ailments on the same setting. To the best of our knowledge, this represents the first case to be reported from Nepal. Worldwide, the leading causes of morbidity and mortality are both AMI and AIS [1]. Both of them have a narrow therapeutic time window and therefore, are considered medical emergencies [4]. Similarly, the annual incidence of AMI after AIS is highest in the first year after the index event (1.1%), followed by a much lower annual risk in the second to fifth years (between 0.16% and 0.27%) [6]. The co-occurrence of AMI and AIS was first described by Omar et al. in 2010 as CCI syndrome [5]. There are three types of CCI, i.e., Type 1: concurrent CCI; Type 2: AIS after recent (<3 months) myocardial infarction; and Type 3: AMI after recent (<3 months) AIS [7]. In our case, the patient developed symptoms of AIS two hours after the onset of chest pain. The key underlying pathogenesis of CCI may be attributed to the atherosclerotic process that may occur simultaneously in both coronary and cerebral arteries, or due to an embolic phenomenon from a cardiac chamber to the cerebral vessels.

The diagnosis of CCI is made by the presence of simultaneous acute onset of a focal neurological deficit, indicating acute stroke, and chest pain with evidence of elevated cardiac enzymes and ST-segment elevation in the ECG, indicating AMI. The incidence of CCI is a rare phenomenon with an incidence of 0.009%, and therefore, the studies regarding it are few and far between [2]. The common causes of CCI are atrial fibrillation, acute aortic dissection, left ventricular thrombus, prosthetic valve thrombosis, and right ventricular thrombus with patent foramen ovale [7].

The dilemma always exists as to which condition needs an earlier intervention. The primary percutaneous coronary intervention (PPCI) for AMI, if chosen as the first management strategy, would delay the management of AIS and would eventually place the patient outside of the therapeutic window for thrombolysis of stroke. Likewise, if the patient undergoes a mechanical thrombectomy for stroke, it would further delay the management of AMI, which might lead to additional myocardial damage, arrhythmias, heart failure, and eventually cardiogenic shock. To add to it, the mandatory requirement of anticoagulants during PPCI and the dual antiplatelet therapy after PPCI imparts a higher risk for the development of intracerebral hemorrhage.

The 2018 guidelines from the American Heart Association/American Stroke Association recommend that in patients presenting with concurrent AMI and AIS without contraindications to thrombolysis and who are hemodynamically stable, IV alteplase at the dose appropriate for cerebral ischemia followed by PCI and stenting for ST-elevation myocardial infarction (STEMI) is reasonable. The therapeutic time window for alteplase is within 4.5 hours of clearly defined symptom onset time, or within 4.5 hours of the time the patient was last known to be well. In contrast, patients with stroke-related to large vessel occlusion who are hemodynamically unstable and who also have contraindications to thrombolysis should be rather treated with mechanical thrombectomy as a part of the initial stroke management [8]. In our case, mechanical thrombectomy was performed for two reasons: (1) the presentation was outside the therapeutic time window for thrombolysis and (2) the patient was in cardiogenic shock. The STEMI part was then dealt with PPCI for the reason that the patient had presented within the therapeutic time window.

## 4. Conclusion

A simultaneous occurrence of AMI and AIS is an extremely rare entity and a poorly studied phenomenon that carries a grave prognosis if not treated promptly. The mode and sequence of revascularization for both entities should be quickly decided without placing the patient outside of the therapeutic time window of intervention. An interventional cardiologist and an interventional neuroradiologist both have a crucial role to play for the effective management of this emergency condition.

## Data Availability

All relevant data supporting the conclusions of this article are included within the article.

## Abbreviations

AMI: Acute myocardial infarction
AIS: Acute ischemic stroke
CCI: Cardio-cerebral infarction
TIMI: Thrombolysis in myocardial infarction
TICI: Thrombolysis in cerebral infarction.

## Supplementary Materials

Supplementary Materials CARE checklist.docx
